# Comparison of the Efficacies and Safety of Combined Therapy between Telbivudine Plus Adefovir and Lamivudine Plus Adefovir in Patients with Hepatitis B Virus Infection in Real-World Practice

**DOI:** 10.1371/journal.pone.0165416

**Published:** 2016-11-02

**Authors:** Ming-Tsung Lin, Yi-Hao Yen, Ming-Chao Tsai, Po-Lin Tseng, Kuo-Chin Chang, Cheng-Kun Wu, Tsung-Hui Hu

**Affiliations:** Division of Hepato-Gastroenterology, Department of Internal Medicine, Kaohsiung Chang Gung Memorial Hospital and Graduate Institute of Clinical Medical Sciences, College of Medicine, Chang Gung University, Taiwan; Yonsei University College of Medicine, REPUBLIC OF KOREA

## Abstract

**Background and Aim:**

Chronic hepatitis B infection remains a significant health issue worldwide. This study evaluated the efficacy and safety of combined therapy using lamivudine plus adefovir (LAM+ADV) versus telbivudine plus adefovir (LdT+ADV) and the corresponding renal function change and safety.

**Methods:**

This study enrolled a total of 171 patients (110 patients received LAM+ADV and 60 patients received LdT+ADV). We analyzed the changes in renal function using the estimated glomerular filtration rate (eGFR). The DNA undetectable rate, hepatitis B e antigen (HBeAg) seroconversion rate, and alanine aminotransferase (ALT) normalization rate were analyzed. We checked the serum uric acid, phosphate and creatine kinase, and lactic acid levels to analyze safety. We observed these patients for 48 to 240 weeks and checked their serum profile every 6 months.

**Results:**

There was no statistically significant difference between the two groups in anti-hepatitis B virus (HBV) efficacy in terms of DNA undetectable rate, ALT normalization rate, and HBeAg seroconversion rate. Both the LAM+ADV and LdT+ADV groups had stable or improved renal function. However, a higher eGFR was found in the LdT+ADV group with continuous serum fluctuation during 3 years of combined therapy as well as a higher serum creatine kinase level.

**Conclusions:**

Long-term LdT+ADV combined therapy and LAM+ADV combined therapy were both associated with stable or improved renal function. The clinical efficacy was similar between the two groups, but the LdT group had a higher serum creatine kinase level. We need to monitor the data regularly in clinical practice.

## Introduction

Chronic hepatitis B virus (HBV) infection remains a major cause of liver-related morbidity and mortality world as well as more than 350 million chronic infections [1,2]. High levels of HBV DNA and detectable hepatitis B e antigen (HBeAg) are risk factors for progression to end-stage complications such as decompensated cirrhosis and hepatocellular carcinoma (HCC) [3, 4]. Treatment of hepatitis B with nucleoside/nucleotide analogues (NUCs) has been shown to reverse fibrosis and cirrhosis and reduce the risk of hepatic decompensation and HCC [5, 6]. Many new antiviral treatments for hepatitis B have become available in the past years, including peginterferon alfa-2a, lamivudine (LAM), adefovir (ADV), entecavir (ETV), and tenofovir (TDF). LAM is the first NUC, and it is a potent inhibitor of HBV replication, which works by competitive inhibition of the reverse transcriptase, and has an excellent safety profile in both compensated and decompensated cirrhotic patients [7]. Meanwhile, LdT is an oral l-nucleoside with minimal hepatic metabolism and is primarily eliminated by renal clearance [8]. However, the disadvantage of LAM and LdT is their very high resistant rate [9, 10]. More than 60% of patients develop LAM resistance within 5 years of treatment, and up to 20% develop LdT resistance within 2 years of treatment [11, 12]. Adefovir dipivoxil (ADV) inhibits HBV DNA polymerase. Clinically, it has a good activity against LAM-resistant HBV strains [13]. However, renal impairment is frequent after long-term treatment with ADV [14, 15]. Hypophosphatemia is another reported side effect [16]. A previous study suggested that in patients with decompensated cirrhosis, LAM+ADV combined therapy could be used as an initial treatment to achieve rapid suppression of HBV replication and reduce the risk of resistance [17]. Moreover, LdT+ADV combined therapy had a good effect in normalizing alanine aminotransferase (ALT) levels, virus clearance, and HBeAg seroconversion in patients with LdT-associated virologic breakthrough or genotypic resistance to LdT [18]. These oral antiviral agents are all eliminated through the renal route [19]. Therefore, in patients with poor renal function, dose reduction or increased dose intervals are recommended [8]. In patients with compensated and decompensated cirrhosis, long-term LdT therapy improved renal function, especially in patients with increased risk for renal impairment [20, 21]. The study of Perrella et al [22] also showed that LdT prophylaxis for HBV was safe and effective without any significant side effect on the liver or on renal function tests after liver transplantation. However, a decrease in the estimated glomerular filtration rate (eGFR) has been observed in patients with chronic hepatitis B under long-term TDF, ADV, or ETV treatment [14, 23]. A previous study also showed that patients with chronic hepatitis B who were treated with LdT exhibited significantly greater virologic and biochemical responses compared with those treated with LAM [24]. Renal impairment is more commonly associated with ADV treatment than with other HBV NUCs [25, 26]. Some symptoms and side effects such as reversible myopathy, neuropathy, and Fanconi syndrome have been reported with LAM treatment [27–29]. Fanconi syndrome is a disease of the proximal renal tubules, with presentation of hypophosphatemia, hypouricemia, etc [30]. LdT has been associated with myopathy and increase in serum creatine kinase levels [31]. Creatine kinase, lactate dehydrogenase, and aspartate aminotransferas are all useful serum markers of muscle injury [32]. The increase in serum creatine kinase was 9% in the LdT treatment and 3% in the LAM treatment^10^. Myalgia and nervous system symptoms and other side effects were also reported after LdT treatment [33]. However, there are no studies comparing renal function and the efficacies and side effects of LdT+ADV treatment versus LAM+ADV treatment, especially in LAM- or LdT-resistant patients. Moreover, the efficacy and safety of combined therapy with LdT+ADV and LAM+ADV in patients with chronic hepatitis B and are not well established. As a result, we aimed to investigate the long-term renal outcome of previously resistant patients treated with LAM+ADV and LdT+ADV. Moreover, we evaluated the virology response and renal function and analyzed the fluctuation of serum phosphate, uric acid, lactic acid, and creatine phosphokinase after combined therapy every 6 months until the end of treatment and compared these biochemical changes with abnormal findings found after monotherapy in previous studies [19,34].

## Materials and Methods

This retrospective study enrolled 171 patients with chronic hepatitis B treated with LdT+ADV and LAM+ADV because of LAM or LdT resistance at the Chang Gung Memorial Hospital, Kaohsiung Medical Center, for at least 1 year (May 2003 to May 2013). The criterion of the National Health Insurance of Taiwan for LAM+ADV or LdT+ADV combination therapy was resistance to LAM or LdT monotherapy. The definition of resistance is >1 log (IU/mL) elevated HBV DNA during LdT or LAM monotherapy. The medication dosages were used according to the manufacturer’s recommendation based on the patients’ renal function. In general, patients took LdT 600 mg plus ADV 10 mg daily or LAM 100 mg plus ADV 10 mg daily. We excluded those patients who received combined therapy less than 1 year or those who have concomitant hepatitis C virus (HCV) or human immunodeficiency virus (HIV) infection. A total of 171 patients were enrolled in this study. In this study, 61 received LdT+ADV therapy as an experimental group. The other 110 received LAM+ADV for comparison. In the LAM plus ADV group, the median duration of LAM monotherapy were 92.5 weeks. In the LdT plus ADV group, the median duration of LAM and LdT monotherapy were 71 weeks and 73 weeks. Serum ALT, phosphate and creatine phosphokinase, uric acid, and lactic acid were routinely measured every 6 months by an automatic analyzer according to manufacturer instructions. We analyzed the fluctuation in renal function using the eGFR, which we calculated every 6 months using the Modification of Diet in Renal Disease (MDRD) equation. We divided these patient into eGFR <60, 60–90, and >90 mL/min/1.73 m^2^ after referring to the KDOQI Clinical Practice Guidelines for Chronic Kidney Disease [35]. We compared the eGFR change after combined therapy with the baseline eGFR. We compared the stage change of eGFR at 48, 96, 144, 192, and 240 weeks. Regarding the antiviral efficacies, we analyzed the DNA undetectable rate, ALT normalization, and HBeAg serological response. The serum DNA level was estimated at baseline and every 6 months until the stop of medication or at end of the study. Serum HBV DNA was assessed quantitatively by real-time TaqMan PCR technology (Roche, Switzerland), with an estimated lower limit of detection of 20 IU/mL. The study protocol conformed to the ethical guidelines of the Declaration of Helsinki and was approved by the Institutional Review Board.

### Statistical analysis

Quantitative data were presented as median (range), and categorical data were expressed as numbers and percentages. The HBV DNA levels were logarithmically transformed for analysis. The comparison of patients with various categories was done using χ^2^ test or Fisher exact test for categorical variables. The continuous variable was analyzed with Student t test. Many data were missing in our study because of the unavailability of preserved blood. Therefore, we used repeated measures and mixed models for statistical analysis. *P* values less than 0.05 were considered statistically significant.

## Results

**Fig 1:**
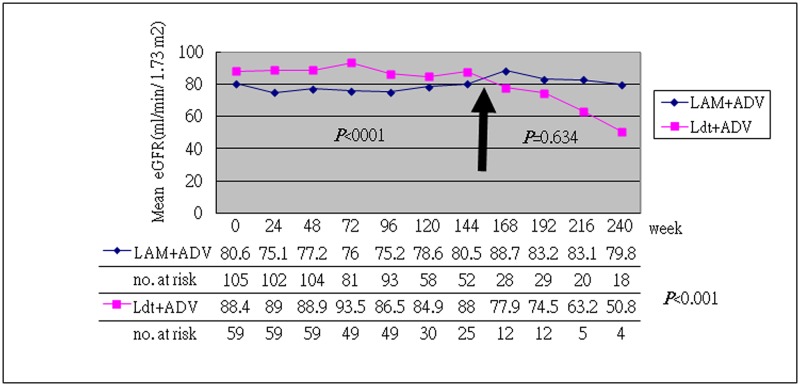
Continuous change in serum eGFR during 240 weeks of combined therapy. During 144 weeks of combined therapy, the LdT+ADV group had a higher eGFR than the LAM+ADV group (*P*<0.001), but both had similar baseline data (*P*>0.05). After 144 weeks of combined therapy, there was no statistically significant difference between the two groups (*P* = 0.634), but there were fewer patients.

**Fig 2:**
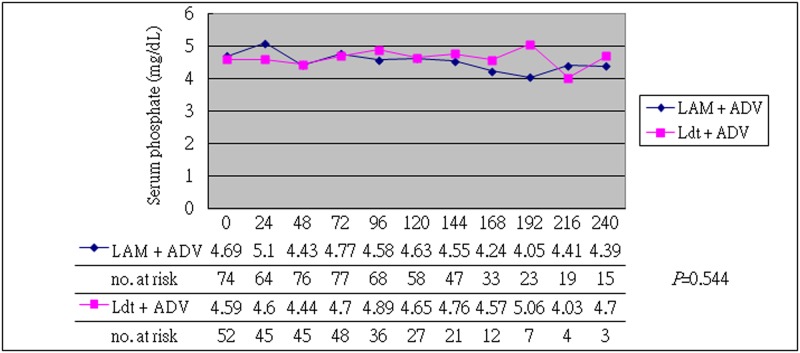
Continuous change in serum phosphate concentration during 240 weeks of combined therapy. There was no statistically significant difference between the two groups (*P* = 0.544).

**Fig 3:**
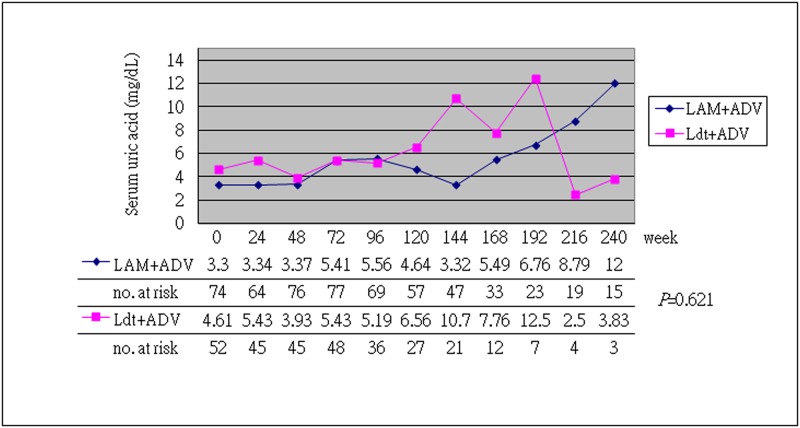
Continuous change in serum uric acid concentration during 240 weeks of combined therapy. There was no statistically significant difference between LAM+ADV and LdT+ADV combined therapy (*P* = 0.621).

The baseline characteristics of the enrolled patients are shown in Table 1. The general baseline characteristics were similar between the two groups (*P*>0.05). The mean baseline HBeAg status, DNA level, serum phosphate, uric acid, lactic acid, and eGFR were similar between these two groups, but the LAM+ADV group had more cirrhotic patients and higher baseline ALT level (*P*<0.05). The mean underlying HBV DNA was 4.98 (3.61–5.97) (log DNA, median (IQR)) IU/ mL in the LAM+ADV group and 4.69 (3.72–6.82) (log DNA, median (IQR)) IU/ mL in the LdT+ADV group (*P*>0.05). A higher baseline serum creatine kinase level was noted in the LdT+ADV group (borderline significant, *P* = 0.045). After 48 to 240 weeks of treatment, both the LdT+ADV and the LAM+ADV groups showed improved renal function, regardless of the duration (48, 96, 144, 192 and 240 weeks) of the combined therapy (Table 2 and S1 to S4 Tables, *P*>0.05). (Notably, the LdT+ADV group had a higher proportion of stable or improved eGFR at every stage of therapy. On the contrary, the LAM+ADV group had a higher proportion of worse eGFR, but with borderline insignificance.) In addition, the LdT+ADV group exhibited a higher mean eGFR than the LAM+ADV group (*P*<0.001) during 144 weeks of combined therapy (Fig 1), but they had similar baseline data. After 144 weeks of combined therapy, there was no statistically significant difference between the two groups (*P* = 0.634), but the number of cases decreased during the follow-up. In terms of efficacy analysis, the ALT normalization rate was 62.3% in the LAM group and 45.2% in the LdT group after 48 weeks of treatment (*P*>0.05, Table 3). After 96 weeks of combined therapy, the ALT normalization rate was 80.6% in the LAM group and 60.9% in the LdT group (*P*>0.05). A similar ALT normalization rate was found in both groups. The HBV DNA undetectable rate was 53.8% after 48 weeks of treatment and 66.7% after 96 weeks of treatment in the LAM group, but 48.6% after 48 weeks of treatment and 52.9% after 96 weeks in the LdT group. A similar HBV DNA undetectable rate was found in both groups as well (*P*>0.05). The HBV e-seroconversion after 48 weeks of treatment was 20% in the LAM group and 13.6% in the LdT group (*P*>0.05). We found a similar antiviral efficacy in both groups. In terms of safety analysis of combined therapy, we found a similar continuous change in the serum phosphate, uric acid, and lactate levels (Figs 2 and 3 and S1 and S3 Figs, *P*>0.05). However, the LdT group had a higher mean serum creatine kinase level than the LAM group (S2 Fig, *P*<0.001), but treatment was not discontinued in any patient because of adverse effect.

**Table 1:** Basic characteristics of 171 combined therapy patients.

	LAM+ADV (n = 110)	LdT+ADV (n = 61)	*P* value
Sex (male/female)	89/21	43/18	0.120
Age (years), median (IQR)	53 (44.75–60.00)	53 (44.00–62.00)	0.833
Liver cirrhosis (yes/no)	51/59	18/43	**0.031**
Treatment duration (weeks), median (IQR)	152 (104.75–191.25)	110 (85–155.5)	**0.003**
HBeAg (+/−)	36/74	26/35	0.197
Baseline eGFR (mL/min/1.73 m^2^), median (IQR)	78 (66–94)	84 (70–101)	0.137
Baseline ALT, U/L, median (IQR)	90.5 (37.5–225.25)	40 (29–92)	**0.002**
Underlying HBV DNA (log DNA) IU/mL, median (IQR)	4.98 (3.61–5.97)	4.69 (3.72–6.82)	0.394
Baseline serum phosphate (mg/dL), median (IQR)	4.5 (4.1–5.33)	4.5 (3.7–5.3)	0.583
Baseline serum CPK (U/L), median (IQR)	76 (52–102)	93 (64–144.75)	**0.045**
Baseline serum uric acid (mg/ dL), median (IQR)	3.1 (2.3–4.15)	3.15 (2.33–4.38)	0.508
Baseline serum lactate (mg/ dL)	65 (47.3–72.6)	57.6 (50.5–70.7)	0.439
Mean baseline serum calcium (mg/dL), median (IQR)	9 (8.55–9.20)	9.3 (8.90–9.50)	**0.021**
Mean baseline BUN/Cr level, median (IQR)	14.49 (11.48–18.33)	15.15 (11.8–22.64)	0.156

IQR, interquartile range; CPK, creatine kinase.

**Table 2:** Changes in eGFR (mL/min/1.73 m^2^) in the LAM+ADV and LdT+ADV treatment groups after 48 weeks of combined therapy.

	Patient number according to eGFR after 48 weeks of treatment			
	<60	60–90	>90	Total
**LAM+ADV group**				
Patient number according to eGFR of baseline, n = 99				
<60	5	8	0	13
60–90	12	38	8	58
>90	1	13	14	28
Improved eGFR, patient number/total (%)	16/99 (16.2)			
Stable eGFR, patient number/total (%)	57/99 (57.6)			
Stable or improved eGFR, patient number/total (%)	73/99 (73.7)			
Decreased eGFR, patient number/total (%)	26/99 (26.3)			
**LdT+ADV group**				
Patient number according to eGFR of baseline, n = 59				
<60	6	2	0	8
60–90	3	17	6	26
>90	0	7	18	25
Improved eGFR, patient number/total (%)	8/59 (13.6)			
Stable eGFR, patient number/total (%)	41/59 (69.5)			
Stable or improved eGFR, patient number/total (%)	49/59 (83.1)			
Decreased eGFR, patient number/total (%)	10/59 (16.9)			
**LAM+ADV versus LdT+ADV group in stable or improved eGFR**				
73/99 versus 49/59, *P* = 0.177				

**Table 3:** Comparison of the efficacies between LAM+ADV and LdT+ADV treatment in ALT normalization rate and DNA undetectable rate and serum HBeAg seroconversion rate.

	LAM+ADV group	LdT+ADV group	*P* value
**ALT normalization rate (<40 U/L) (yes/total) (%)**			
48 weeks	48/77 (62.3)	14/31 (45.2)	0.102
96 weeks	58/72 (80.6)	14/23 (60.9)	0.055
**DNA undetectable rate (<20 IU/mL) (yes/total) (%)**			
48 weeks	21/39 (53.8)	17/35 (48.6)	0.650
96 weeks	30/45 (66.7)	9/17 (52.9)	0.318
**e-Seroconversion rate (yes/total) (%)**			
within 48 weeks	6/30 (20)	3/22 (13.6)	0.717

## Discussion

This study comprehensively compared the efficacy and safety of LdT+ADV and LAM+ADV combined therapy, which is important because chronic hepatitis B remains a public health problem despite the advancements in medicine. End-stage liver disease secondary to HBV is the leading indication for liver transplantation in Asia [36]. Studies [37, 38] showed that the risk for HCC was greatest in those who were older and who remained HBeAg-positive and increased significantly in individuals with a high serum HBV-DNA level. As a result, the normalization of liver enzyme levels, viral suppression and clearance, and reduction in histologic scores of liver inflammation or fibrosis are important for HBV treatment. The loss of HBsAg or HBeAg seroconversion is the optimal goal of treatment and is associated with a lower incidence of cirrhosis and HCC and improved survival rates [39–41]. A previous study showed that in patients who did not respond to LAM monotherapy, LAM+ADV combined therapy could increase ALT normalization and HBV DNA clearance, but not HBeAg clearance or seroconversion [42]. The study of Yao et al [43] also showed that LdT+ADV combination treatment had better efficacy than LdT monotherapy and lower drug resistance rate in chronic hepatitis B patients with higher viral load. Chen et al [44] had the similar conclusion that prolonged combination therapy (LAM+ADV and LdT+ADV) was more effective than monotherapy (ADV), regardless of the ALT normalization rate, sustained viral response, or resistance rate. Our previous study, despite the smaller number of patients, demonstrated that LdT+ADV is a suitable second-line salvage therapy for LAM-resistant patients [45]. In our present study, we found the similar effect in terms of HBeAg seroconversion, HBV DNA undetectable rate, and/or ALT normalization rate in the LdT+ADV and LAM+ADV groups after 48 or 96 weeks of treatment (*P*<0.05). The M204 V/I or L180M signature mutations were the primary basis for LdT and LAM resistance in past studies and in our patient groups, but these mutations remained sensitive to ADV treatment [46,47]. As a result, we found similar treatment efficacies between the two groups. The study of Gane et al [20] noted a sustained improvement in renal function in the LdT treatment group but not in the LAM group; however, in our study, we found that renal function improved in both the LdT+ADV group and the LAM+ADV group after 48 to 240 weeks of combined therapy (*P>*0.05). However, the LdT+ADV group had a higher mean eGFR during 144 weeks of treatment (*P*<0.05), which is compatible with a previous monotherapy study [14], which also showed that ADV monotherapy may impair renal function. Furthermore, although TDF had better antiviral potency than LdT and is the newest medication for hepatitis B recommended by the American Association for the Study of Liver Diseases (AASLD), LdT was found to have a more superior renal protection than TDF [48, 49]. The mechanism of improvement in renal function after combined therapy remains to be determined but may be related with HBV eradication. Liaw et al [12] showed that the safety profile of LdT is similar to LAM, but elevated serum creatine kinase levels were more common in patients under LdT treatment. Our study found the similar phenomenon in combined therapy. Lin et al [50] concluded that LdT has a renoprotective effect when HBV patients under cisplatin-based chemotherapy for HCC receives LdT preemptive therapy. Gane et al [20] showed that improvement in eGFR was observed in the LdT group but not in the LAM group. We noted the various conclusions regarding renal function from different previous studies. However, in our long-term follow-up on the fluctuation of renal function after combined therapy to 5 years, we found more patients with improved renal function, with a similar proportion between patients who received LdT+ADV and LAM+ADV combined therapy (*P*>0.05). LdT treatment can induce substantial myopathy. As a result, we need to monitor the muscular function and clinical symptoms of the patients treated with this drug to prevent possible major complications. In our present study, we validated the similar phenomenon in combined therapy. However, no significant difference was found in other parameters such as serum phosphate, uric acid, or lactate. Though we clarified the benefit and side effect of combined therapy (LAM+ADV or LdT+ADV), however, add-on ADV is no longer the treatment options nowadays. The newest AASLD guideline suggest that tenofovir and entecavir are preferred because of their better potency and lower risk of resistance [51]. As a result, finally, in the LAM+ADV group (110 patients), 47 patients (42.7%) switched to TDF and in the LdT+ADV group (61 patients), 24 patients (39.3%) switched to TDF in our study.

In conclusion, our study provided clinical evidence that combination therapy with LdT+ADV or LAM+ADV leads to significant decreases in serum HBV DNA and ALT levels, without specific damage to renal function in chronic hepatitis B patients.

Our study showed a similar proportion of renal function improvement between the LAM+ADV and the LdT+ADV groups during 48 to 240 weeks of combined therapy but higher mean eGFR in the LdT group during treatment. We also provided a valuable safety database for combined therapy for clinical practice, as combined therapy continues to be provided to some patients with LdT or LAM resistance.

## Supporting Information

S1 Fig Continuous change in serum lactate concentration during 240 weeks of combined therapy. There was no statistically significant difference between LAM+ADV and LdT+ADV combined therapy (P = 0.728).
(TIF)

S2 Fig Continuous change in serum creatine kinase (CPK) concentration among 240 weeks combined therapy. Higher serum CPK concentrations were found in the LdT+ADV group during 240 weeks of combined therapy (P<0.001).
(TIF)

S3 Fig Combined analysis of continuous change in serum eGFR and phosphate concentration among 240 weeks combined therapy. (TIF)

S1 Table Changes in eGFR (mL/min/1.73 m^2^) in the LAM+ADV and LdT+ADV treatment groups after 96 weeks of combined therapy. (DOC)

S2 Table Changes in eGFR (mL/min/1.73 m^2^) in the LAM+ADV and LdT+ADV treatment groups after 144 weeks of combined therapy. (DOC)

S3 Table Changes in eGFR (mL/min/1.73 m^2^) in the LAM+ADV and LdT+ADV treatment groups after 192 weeks of combined therapy. (DOC)

S4 Table Changes in eGFR (mL/min/1.73 m^2^) in LAM+ADV and LdT+ADV treatment after 240 weeks of combined therapy. (DOC)
