# Supplementary figures and images for: Comparison of the Efficacies and Safety of Combined Therapy between Telbivudine Plus Adefovir and Lamivudine Plus Adefovir in Patients with Hepatitis B Virus Infection in Real-World Practice
Source: PLoS One. 2016 Nov 2;11(11):e0165416. doi: 10.1371/journal.pone.0165416 (PMC5091898; doi:10.1371/journal.pone.0165416)

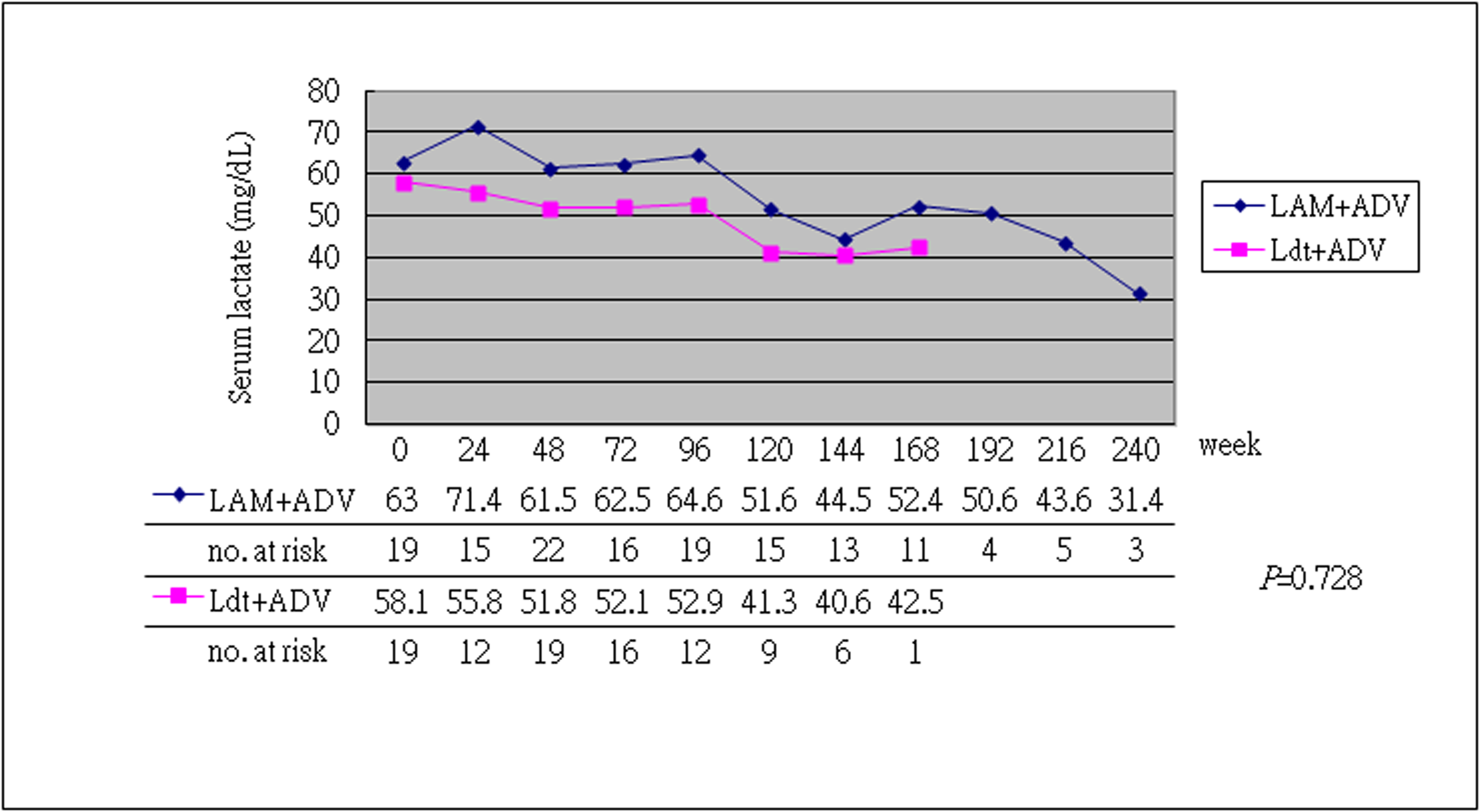

Supplement: S1 Fig — There was no statistically significant difference between LAM+ADV and LdT+ADV combined therapy (P = 0.728). (TIF) [file pone.0165416.s001.tif]

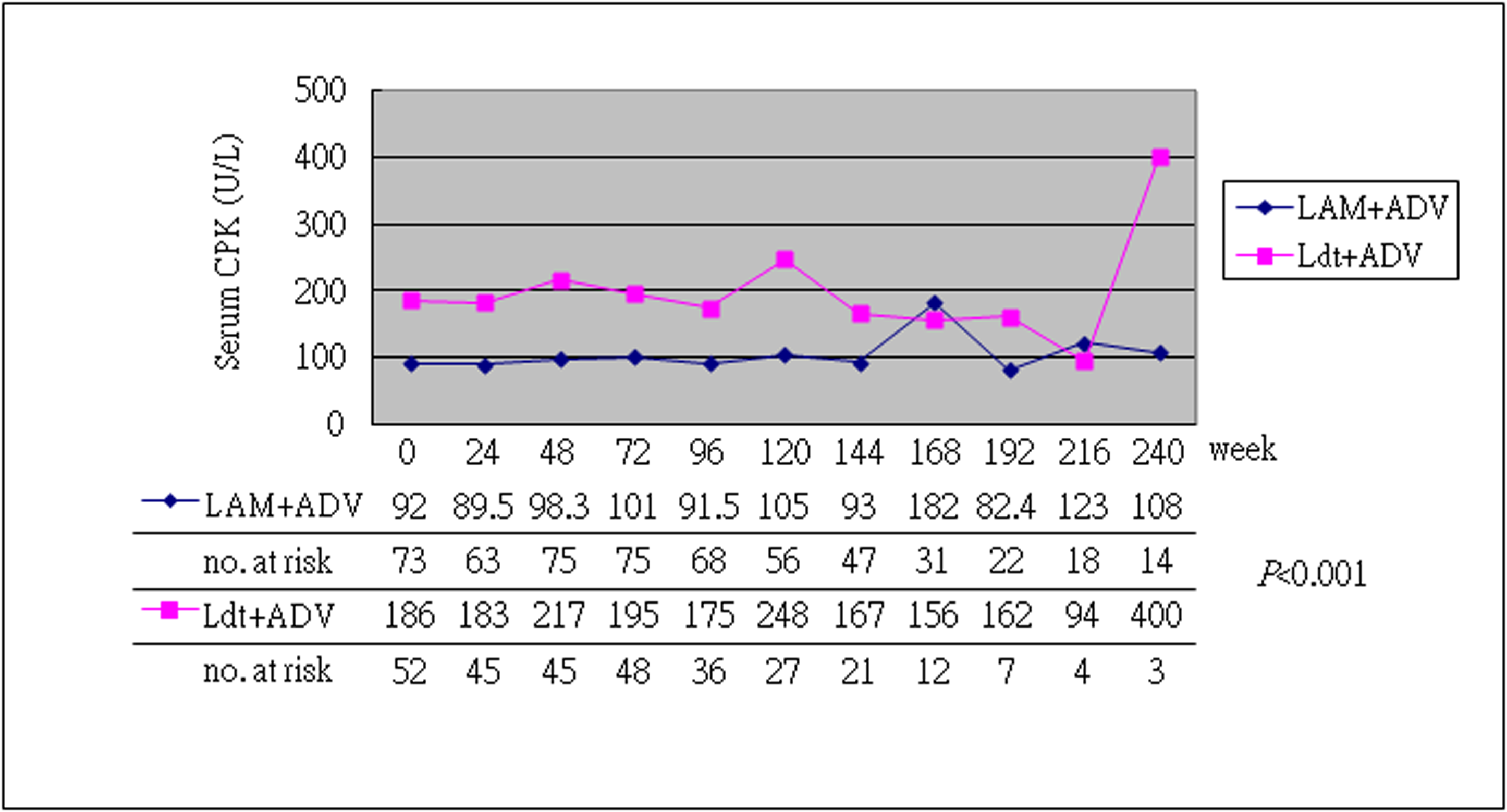

Supplement: S2 Fig — Higher serum CPK concentrations were found in the LdT+ADV group during 240 weeks of combined therapy (P<0.001). (TIF) [file pone.0165416.s002.tif]

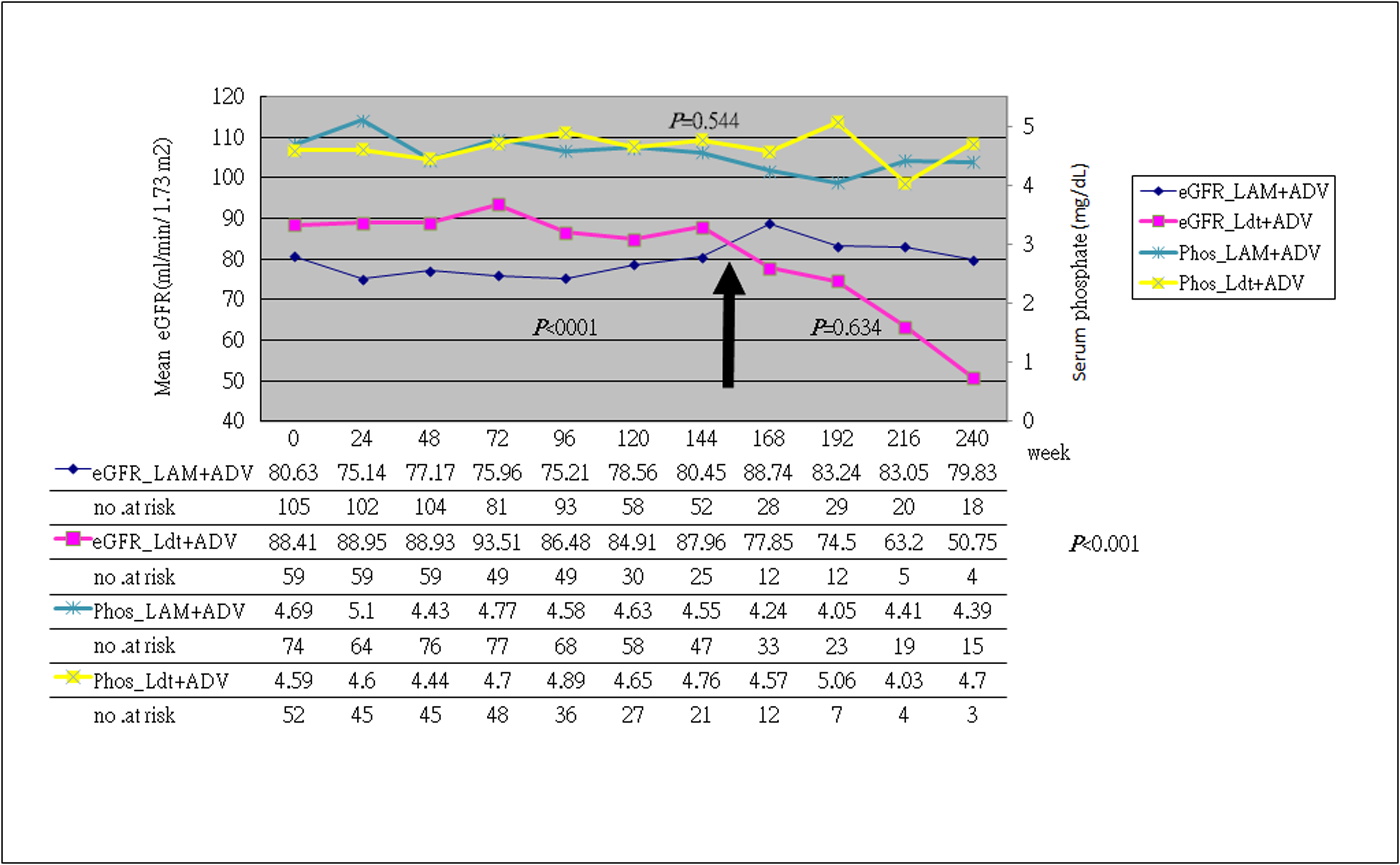

Supplement: S3 Fig — (TIF) [file pone.0165416.s003.tif]
