# Supplementary material for: Comparison of the Efficacies and Safety of Combined Therapy between Telbivudine Plus Adefovir and Lamivudine Plus Adefovir in Patients with Hepatitis B Virus Infection in Real-World Practice
Source: PLoS One. 2016 Nov 2;11(11):e0165416. doi: 10.1371/journal.pone.0165416 (PMC5091898; doi:10.1371/journal.pone.0165416)
Supplement: S1 Table — (DOC) [file pone.0165416.s004.doc]

**Supplementary Table 1.** Changes in eGFR (mL/min/1.73 m^2^) in the LAM+ADV and LdT+ADV treatment groups after 96 weeks of combined therapy

|  | Patient number according to eGFR after 96 weeks of treatment | | | |
| --- | --- | --- | --- | --- |
|  | <60 | 60-90 | >90 | Total |
| **LAM+ADV group** | | | | |
| Patient number according to eGFR of baseline (n=86) | | | | |
| <60 | 10 | 3 | 0 | 13 |
| 60-90 | 12 | 33 | 4 | 49 |
| >90 | 1 | 11 | 12 | 24 |
| Improved eGFR, patient number/total (%) | 7/86 (8.1) | | | |
| Stable eGFR, patient number/total (%) | 55/86 (64) | | | |
| Stable or improved eGFR, patient number/total (%) | 62/86 (72.1) | | | |
| Decreased eGFR, patient number/total (%) | 24/86 (27.9) | | | |
| **LdT+ADV group** | | | | |
| Patient number according to eGFR of baseline (n=49) | | | | |
| <60 | 5 | 4 | 0 | 9 |
| 60-90 | 3 | 9 | 9 | 21 |
| >90 | 0 | 5 | 14 | 19 |
| Improved eGFR, patient number/total (%) | 13/49 (26.5) | | | |
| Stable eGFR, patient number/total (%) | 28/49 (57.1) | | | |
| Stable or improved eGFR, patient number/total (%) | 41/49 (83.7) | | | |
| Decreased eGFR, patient number/total (%) | 8/49 (16.3) | | | |
| **LAM+ADV versus LdT+ADV group in stable or improved eGFR** | | | | |
| 62/86 versus 41/49, *P*=0.128 | | | | |
