# Supplementary material for: Comparison of the Efficacies and Safety of Combined Therapy between Telbivudine Plus Adefovir and Lamivudine Plus Adefovir in Patients with Hepatitis B Virus Infection in Real-World Practice
Source: PLoS One. 2016 Nov 2;11(11):e0165416. doi: 10.1371/journal.pone.0165416 (PMC5091898; doi:10.1371/journal.pone.0165416)
Supplement: S2 Table — (DOC) [file pone.0165416.s005.doc]

**Supplementary Table 2.** Changes in eGFR (mL/min/1.73 m^2^) in the LAM+ADV and LdT+ADV treatment groups after 144 weeks of combined therapy

|  | Patient number according to eGFR after 144 weeks treatment | | | |
| --- | --- | --- | --- | --- |
|  | <60 | 60-90 | >90 | Total |
| **LAM+ADV group** | | | | |
| Patient number according to eGFR of baseline (n=46) | | | | |
| <60 | 3 | 2 | 0 | 5 |
| 60-90 | 5 | 16 | 6 | 27 |
| >90 | 0 | 3 | 11 | 14 |
| Improved eGFR, patient number/total (%) | 8/46 (17.4) | | | |
| Stable eGFR, patient number/total (%) | 30/46 (65.2) | | | |
| Stable or improved eGFR, patient number/total (%) | 38/46 (82.6) | | | |
| Decreased eGFR, patient number/total (%) | 8/46 (17.4) | | | |
| **LdT+ADV group** | | | | |
| Patient number according to eGFR of baseline (n=26) | | | | |
| <60 | 3 | 4 | 0 | 7 |
| 60-90 | 1 | 4 | 6 | 11 |
| >90 | 0 | 1 | 7 | 8 |
| Improved eGFR, patient number/total (%) | 10/26 (38.5) | | | |
| Stable eGFR, patient number/total (%) | 14/26 (53.8) | | | |
| Stable or improved eGFR, patient number/total (%) | 24/26 (92.3) | | | |
| Decreased eGFR, patient number/total (%) | 2/26 (7.7) | | | |
| **LAM+ADV versus LdT+ADV group in stable or improved eGFR** | | | | |
| 38/46 versus 24/26, *P*=0.311 | | | | |
