# Supplementary material for: Comparison of the Efficacies and Safety of Combined Therapy between Telbivudine Plus Adefovir and Lamivudine Plus Adefovir in Patients with Hepatitis B Virus Infection in Real-World Practice
Source: PLoS One. 2016 Nov 2;11(11):e0165416. doi: 10.1371/journal.pone.0165416 (PMC5091898; doi:10.1371/journal.pone.0165416)
Supplement: S3 Table — (DOC) [file pone.0165416.s006.doc]

**Supplementary Table 3.** Changes in eGFR (mL/min/1.73 m^2^) in the LAM+ADV and LdT+ADV treatment groups after 192 weeks of combined therapy

|  | Patient number according to eGFR after 192 weeks treatment | | | |
| --- | --- | --- | --- | --- |
|  | <60 | 60-90 | >90 | Total |
| **LAM+ADV group** | | | | |
| Patient number according to eGFR of baseline (n=28) | | | | |
| <60 | 1 | 1 | 0 | 2 |
| 60-90 | 1 | 13 | 1 | 15 |
| >90 | 0. | 3 | 8 | 11 |
| Improved eGFR, patient number/total (%) | 2/28 (7.1) | | | |
| Stable eGFR, patient number/total (%) | 22/28 (78.6) | | | |
| Stable or improved eGFR, patient number/total (%) | 24/28 (85.7) | | | |
| Decreased eGFR, patient number/total (%) | 4/28 (14.3) | | | |
| **LdT+ADV group** | | | | |
| Patient number according to eGFR of baseline (n=12) | | | | |
| <60 | 2 | 3. | 1 | 6 |
| 60-90 | 2 | 1 | 3 | 6 |
| >90 | 0 | 0 | 0 | 0 |
| Improved eGFR, patient number/total (%) | 7/12 (58.3) | | | |
| Stable eGFR, patient number/total (%) | 3/12 (25) | | | |
| Stable or improved eGFR, patient number/total (%) | 10/12 (83.3) | | | |
| Decreased eGFR, patient number/total (%) | 2/12 (16.7) | | | |
| **LAM+ADV versus LdT+ADV group in stable or improved eGFR** | | | | |
| 24/28 versus 10/12, *P*=1.000 | | | | |
