# Supplementary material for: Comparison of the Efficacies and Safety of Combined Therapy between Telbivudine Plus Adefovir and Lamivudine Plus Adefovir in Patients with Hepatitis B Virus Infection in Real-World Practice
Source: PLoS One. 2016 Nov 2;11(11):e0165416. doi: 10.1371/journal.pone.0165416 (PMC5091898; doi:10.1371/journal.pone.0165416)
Supplement: S4 Table — (DOC) [file pone.0165416.s007.doc]

**Supplementary Table 4**. Changes in eGFR (mL/min/1.73 m^2^) in LAM+ADV and LdT+ADV treatment after 240 weeks of combined therapy

|  | Patient number according to eGFR after 240 weeks treatment | | | |
| --- | --- | --- | --- | --- |
|  | <60 | 60-90 | >90 | Total |
| **LAM+ADV group** | | | | |
| Patient number according to eGFR of baseline (n=16) | | | | |
| <60 | 0 | 0 | 0 | 0 |
| 60-90 | 2 | 6 | 1 | 9 |
| >90 | 0 | 3 | 4 | 7 |
| Improved eGFR, patient number/total (%) | 1/16 (6.3) | | | |
| Stable eGFR, patient number/total (%) | 10/16 (62.5) | | | |
| Stable or improved eGFR, patient number/total (%) | 11/16 (68.75) | | | |
| Decreased eGFR, patient number/total (%) | 5/16 (31.25) | | | |
| **LdT+ADV group** | | | | |
| Patient number according to eGFR of baseline (n=4) |  | | | |
| <60 | 1 | 2 | 0 | 3 |
| 60-90 | 1 | 0 | 0 | 1 |
| >90 | 0 | 0 | 0 | 0 |
| Improved eGFR, patient number/total (%) | 2/4 (50) | | | |
| Stable eGFR, patient number/total (%) | 1/4 (25) | | | |
| Stable or improved eGFR, patient number/total (%) | 3/4 (75) | | | |
| Decreased eGFR, patient number/total (%) | 1/4 (25) | | | |
| **LAM+ADV versus LdT+ADV group in stable or improved eGFR** | | | | |
| 11/16 versus 3/4, *P*=1.000 | | | | |
